# Vaginal-Hysterotomy, and Subsequent Delivery with Forceps with Safety to Both Mother and Child

**Published:** 1848-05

**Authors:** 


*Vaginal-Hysterotomy, and subsequent delivery with forceps, with safety to both mother and child.* By Gunning S. Bedford, M. D. Professor of Midwifery and the diseases of Women and Children, in the University of New York.

[Extracted from the American Journal of the Medical Sciences for April, 1848.]

On Saturday, Nov. 6th, 1847, at 5 o'clock A. M., Doctor Alexander Clinton was summoned to attend Mrs. L., aged 36, in labour with her first child. Dr. Clinton had been for some time the family physician of Mrs. E.. and had attended her in repeated and occasionally severe attacks of nephritis. On reaching the house, he

---

found Mrs. L. in labour, the pains being decided, and occurred with regularity at intervals of fifteen and twenty minutes. In his examination per vaginam, the Doctor was unable to detect the *os tincæ*; he very cautiously explored the vaginia and persenting portion of the womb with his finger ; and after several fruitless attempts to find the mouth of the womb, he came to the conclusion that the difficulty of reaching the *os* was owing to mal-position of the organ —probably retroversion of the cervix. Accordingly, he waited until evening, when the pains increasing in violence, and assuming an expulsive character, he again examined his patient, but with no better success. He then proposed a consultation, the patient having been in labour fourteen hours. My colleague, Professor Valentine Mott, was sent for ; on hearing the particulars of the case, he made a vaginal examination ; and, after repeated attempts, he failed in finding the mouth of the womb. Professor Mott suggested that possibly some change would occur during the night in the position of the parts, which would enable him to reach the *os uteri*, and left the house with the promise that he would return in the morning. Doctor Clinton continued with his patient during the night, and the pains occurred regularly with more or less force. He made several examinations in the night, and could find nothing but a globular, smooth, and uniform surface. In the morning, Nov. 7th, at ten o'clock, Prof. Mott returned. The pains were then much more violent and the patient suffered severely. He again attempted by examination to reach the mouth of the womb—and again failed. To use his own language, "I have seen a great many obstetric cases, and have attended almost every variety of parturition ; but it is the first time, after 36 hours labour, that I could not feel the *os tincæ*." the case was now assuming a dangerous character—the pains were frequent and expulsive, with an obliterated mouth of the womb. The fear, therefore, was rupture of this organ, and death of the patient with but little chance for the life of the child. The husband friends were informed of the precarious situation of the patient. Doctors Mott and Clinton decided to have additional consultation ; and at the request of these gentlemen, I met them at one o'clock on Sunday, the patient having been in more or less active labour for forty hours. On examining the patient I could not feel the slightest trace of an os tincæ ; and I became satisfied, after a thorough exploration, that it was entirely obliterated. Under these circumstances the death of the mother being inevitable without an operation, it was proposed to lay the womb open through the vagina ; and at the request of Drs. Mott and Clinton, I proposed to perform the operation as follows : with a probe-pointed bistoury covered to within a few lines of its extremity with linen, and taking my finger, as a guide, I made a bi-lateral section of the neck of the womb, extending the incision to within a line or two of the peritoneal cavity. The head of the child was immediately felt through the opening. The pain continued with violence, but there was no progress in the delivery—the neck of the womb was extremely hard and resisting, and presented to the touch after the incision, a cartilaginous feel. Dr. Mott and myself then left the patient in charge of Dr. Clinton, and returned again at six o'clock in the evening. At this time, although the pains had been severe, the head had not descended, nor had any impression been made upon the opening. I then made an incision through the posterior lip. The patient was not in a condition to sustain blood-letting, and a weak solution of tartar emetic was administered with a view, if possible of producing relaxation. Dr. Clinton remained with the patient, and promised, if anything occurred during the night, to inform us of it. We were both sent for at two o'clock, the patient suffering severely from violent and excessive pain, all of which produced little or no change on the head of the child. We remained until seven o'clock in the morning, when we left. The patient being much fatigued, a Dover's powder was ordered, which procured a comfortable sleep, and temporary immunity from suffering. We called again at eleven o'clock; the opening had dilated somewhat, and the head could be more distinctly felt, but it had not begun to engage in the pelvis. There was much heat about the parts, and the scalp was corrugated. The pains continued with regularity, losing nothing in violence, and about six o'clock on the evening of Monday, the patient's strength was evidently giving away, and her pulse rose to 140. In a word, the symptoms were most alarming. The question now presented itself, what was to be done? After mature deliberation, being essentially conservative in the whole management of the case, we determined to make an atempt to deliver with the forceps—certainly not an easy thing to do with the head at the superior strait, not begun to engage in the pelvis, and the opening of the womb not larger than a dollar piece, rigid and unyielding. The forceps, however, after a full view of the circumstances, presented to us the most feasible means of effecting delivery. At the request of Doctors Mott and Clinton, I applied the forceps, and was fortunate, without much loss of time, in locking the instrument. The head was situated diagonally on the upper strait with flexion but partially made. At first I directed my traction downwards and backwards, the handle of the forceps forming an acute angle with the axis of the inferior strait of the pelvis, and when I succeeded in flexing the chin of the child upon the sternum, I then rotated the handle of the instrument for the purpose of giving the demi-spiral movement of the head. In this way, after very great effort, I succeeded in bringing the head to the inferior strait, the occiput pressing on the perineum. At this stage of the operation, my arms and hands were nearly paralyzed, such was the force necessary to overcome the difficulty. I requested Dr. Mott, who was by my side, to relieve me, and he, after no inconsiderable effort, succeeded in bringing the head into the world —and our gratification was in no way diminished by the fact that the child was alive, an event certainly not to have been expected. As strange as it may appear, the only inconvenience experienced by the mother after her delivery was an inability to pass her water—this continued about two weeks, rendering it necesssary to introduce the catheter twice, daily, for the purpose of relieving the bladder. The mother and child are in the enjoyment of excellent health, it being now three months since the operation.

It may, perhaps, be thought by some that the patient should have been delivered sooner, and that we subjected her to serious and unnecessary hazard in delaying the delivery by forceps. This reasoning might possibly be sustained on general principles—but, I think it will be conceded that in this individual case, we were not only justified in the delay, but the result proved the wisdom of the course we pursued. In my judgment, nothing, under the peculiar circumstances of the case, could have warranted any attempt at artificial delivery, *save an approach to exhaustion on the part of the mother, or the occurrence of some accident placing life in the most imminent peril.* The position of the fœtal head, and the condition of the mouth of the womb were such as to render extremely probable failure of any attempt at delivery. The obvious indication, therefore, was to trust to nature as long as she was capable of acting, and for the accoucheur to proceed to artificial delivery the moment the general system exhibited evidences of prostration.

This is the second time I have performed the operation of *vaginal-hysterotomy*, and in both instances the lives of both mother and child were saved. The first case occurred in a female, whose womb had been seriously injured in consequence of attempts made to occasion miscarriage by the notorious abortionist *Madame Restell.* The injury inflicted resulted in entire obliteration of the mouth of the womb. The patient was taken in labour, Dec. 18th, 1843, at 7 o'clock, P. M. and was attended by Drs. Vermeule and Holden. On the following day, at 7 P. M. I was requested to see her in consultation with these gentlemen. Her pains were violent, and she suffered intensely. On making an examination, it was quite evident that there was obliteration of the mouth of the womb. In the presence of Drs. Washington, Detmold, Doane, Vermeule, and Holden, I made a bilateral section of the uterus—and in ten minutes after the incision, the patient was delivered of a full-grown living child. The mother and child continued to do well without one untoward symptom.

I am not aware that this operation has ever been performed in America—at least I have found no record of it. A full account of this case was published in the *New-York Journal of Medicine* for March, 1843.

New-York, February 1st, 1848.

[We are promised for our next No. the report of a case in which Vaginal Hysterotomy was performed many years ago, by a physician now residing in this city.—Ed. Buff. Med. Jour.]

